# CAR T Cells: Cancer Cell Surface Receptors Are the Target for Cancer Therapy

**DOI:** 10.34172/apb.2022.051

**Published:** 2021-08-22

**Authors:** Behrouz Shademan, Vahidreza Karamad, Alireza Nourazarian, Cigir Biray Avcı

**Affiliations:** ^1^Department of Medical Biology, Faculty of Medicine, EGE University, Izmir, Turkey.; ^2^Department of Basic Medical Sciences, Khoy University of Medical Sciences, Khoy, Iran.

**Keywords:** CAR-T cell, Immunotherapy, Surface receptors, Tumor microenvironment

## Abstract

Immunotherapy has become a prominent strategy for the treatment of cancer. A method that improves the immune system’s ability to attack a tumor (Enhances antigen binding). Targeted killing of malignant cells by adoptive transfer of chimeric antigen receptor (CAR) T cells is a promising immunotherapy technique in the treatment of cancers. For this purpose, the patient’s immune cells, with genetic engineering aid, are loaded with chimeric receptors that have particular antigen binding and activate cytotoxic T lymphocytes. That increases the effectiveness of immune cells and destroying cancer cells. This review discusses the basic structure and function of CAR-T cells and how antigenic targets are identified to treat different cancers and address the disadvantages of this treatment for cancer.

## Introduction

**Figure 1:**
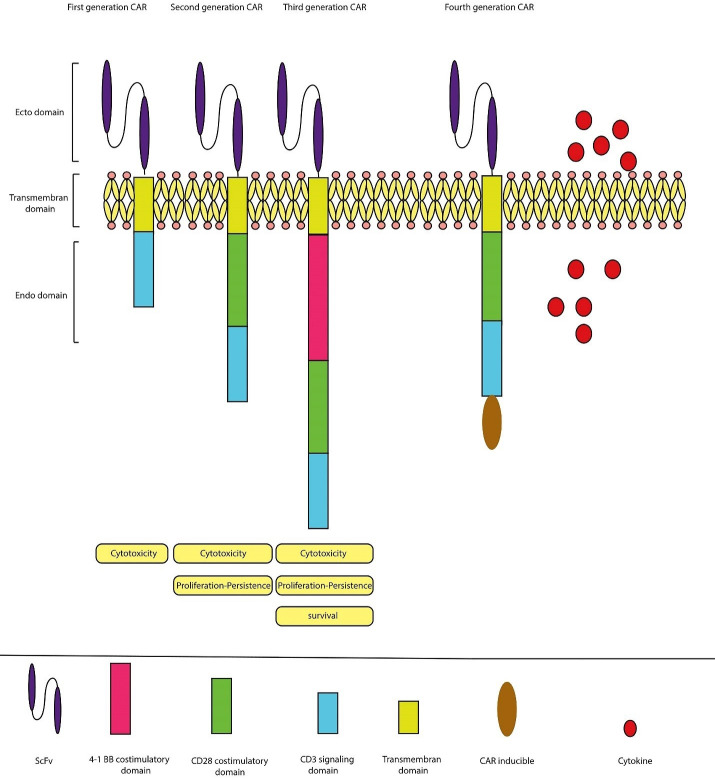
The CAR-T cells are made up of three generations. The first generation of CAR-T cells is composed of scFv and CD3 immunoglobulin. Stimulating molecules such as CD28, CD134, and CD137 (4-1BB) have been attached to CAR-T cells, producing second and third generations.

Cancer is a multi-stage process in a cell for some causes, that begins with DNA damage, unregulated cell proliferation, and apoptosis. Statistics have shown that cancer is the second most severe and deadly disease.^1^ In addition, cancers have the potential to develop resistance to traditional therapies.^2,3^ Therefore It is essential to develop treatment alternatives. However, gene therapy, radiotherapy, chemotherapy, etc. are used to treat cancer. Immunotherapy has recently become the priority of researchers. Strategies that stimulate the immune system to kill tumor cells. Zelig Eshharand and colleagues have developed a hetero-dimer monoclonal antibody for the first time. If these antibodies are transferred to T cells, tumor cells can be detected and killed. In 2008, Malcolm Brenner and Houston made the first successful phase in the therapeutic application of CAR-T cells.^4^ CAR-T cells consist of the target receptor segment (ScFv single-stranded variable fragment attached to the hinge), the membrane-permeable region, and the intracellular region.^5^ The ScFv extracellular antigen recognition fragment was formed of a monoclonal antibody (mAb). That targets specific antigens but can contain ligands that bind to tumor cell surface antigens.^6,7^ Various hinges have been found. These hinges can be short or long. Studies have shown that the hinge is a crucial structure for engineered cells and can significantly boost the efficiency of these cells.^8^ There are structural variations in the intracellular region. Based on differences in intracellular messenger regions, CAR-T cells are categorized into the first, second, third, and fourth generations (Figure 1). The first generation includes the cytoplasmic domain of CD3 ζ, which can initiate T-cell activation. Since the proliferation of T cells is low, they are less effective. Defects of the first generation have been overcome in the second and third generations due to the diversity of co-stimulatory molecules (CMs). This generation is called TRUCK (T cells redirected to universal cytokine killing).^9^ TRUCK is a method of generating a particular CAR-T cell that stimulates an innate immune response to the destruction of tumor cells. These CAR-T cells have an engineered product, including IL-12.^10^

**Figure 2:**
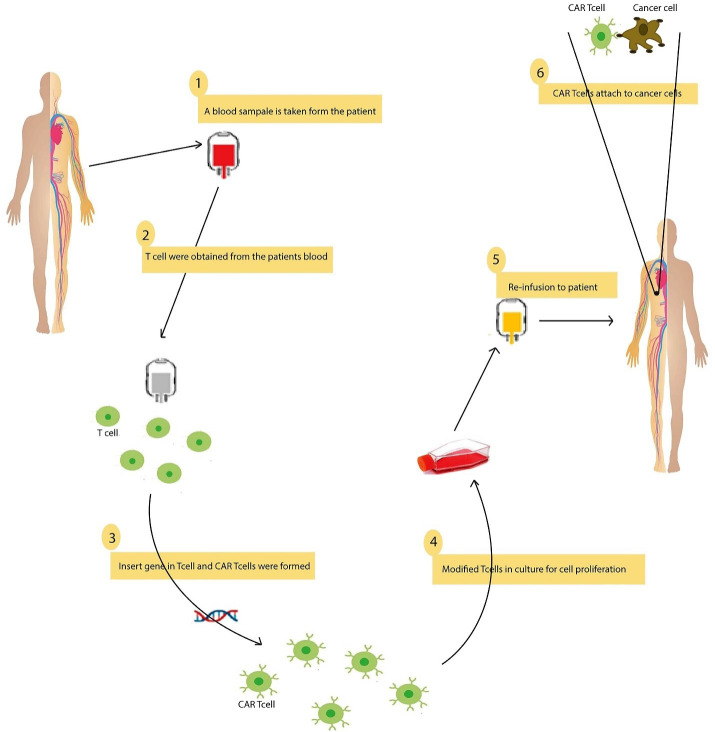
Engineering CAR-T cells for therapeutic purposes. 1) this section begins by obtaining blood from a cancer patient at the hospital. 2) T cells are separated from the blood during this process, and the remaining blood is returned to the body. 3) Following these steps, the T cells are appropriate for genetic modification in the laboratory. Relevant genetic modifications are made in the laboratory on cells isolated from cancer patients. 4) CAR-T cells are culture in the laboratory to produce more cells. 5) The cells produced are eventually transferred to the cancer patient by intravenous injection. 6) Modified T cells now have unique receptors on their surface that are considered chimeric antigen receptors (CAR). These receptors help T cells accurately detect and destroy cancer cells.

The construction of CAR-T cells for therapeutic purposes includes five steps: T cell isolation, T cell activation, T cell engineering, CAR-T cell expansion, cell formulation, and cell storage (Figure 2). Patients have prescribed cells developed for treatment.^11^ The time needed for T cells’ genetic engineering is approximately two weeks.^12,13^ The fourth generation of CAR-T cells performs better in TME than other generations due to their high ability to destroy cancer cells. TME decreases the potency of the immune system. These agents are secreted by tumor cells and are used to defend these cells against immune cells. The TRUCK cells are engineered to act better in TME by secreting inflammatory cytokines.^10^

## Functional mechanism of CAR-T

Cells are designed with specific antigenic receptors that enable modified T cells to acquire more advanced and directed anti-tumor properties. Auxiliary molecules can help T cells better fight cancer cells. Studies have shown that when a signaling molecule called “STAT5” is kept in CAR-T cells, CAR-T cells can stay active longer and help kill the tumor more effectively.^14^ But, there is no requirement for auxiliary molecules to recognize cancer cells.^15^ CAR-T cells can identify antigens of different structures, including proteins, carbohydrates, and glycolipids.^16^ While designed, the genes encoding the CAR structure are transferred to the leukocytes through viral vectors. This intervention should be taken on autologous leukocytes due to the presence of self-identified molecules in T cells. This concern is overcome in patients such as lymphopenia that may not have sufficient lymphocytes to produce genetically engineered donor cells without autoimmune molecules such as class 1 HLA.^17,18^ As CAR-T cells bind explicitly to tumor-associated antigens, T cells are triggered by phosphorylation that leads to the release of cytokines and cancer cells’ death.^19^ Engineered T cells promote cell death through two main pathways: first, the release of perforin and granules, and second, through the triggering of the Fas or TNF signals. Cytotoxic T cells destroy tumor cells in two ways, so while T Helper cells kill cells by perforin/granzyme.^20,21^

## Tumor microenvironment

In solid tumors, the TME consists of various cellular and molecular components that reduce the immune response’s strength against the tumor.^22^ TME prevents the proper function of CAR-T cells by decreasing penetration of CAR-T cells and compromising anti-tumor activity.^23,24^ Cytokines such as TGF-β and IL-10 are developed by TME tumor cells. These agents can suppress a more robust immune response to the tumor and protect cancer cells. T lymphocytes such as TRUCKs are also used in these circumstances. Expressly, CAR-T cells release IL-12 and CAR-T cells, which induce tumor treatment due to micro-environmental tumor disruption.^11^ Cancer cells evade the immune system with the aid of TME so that additional auxiliary agents would be required for long-term responses. Decreased immunosuppressive agents such as Tregs and TAM, enhance treatment efficacy with engineered cells.^25^ Immunosuppressive factors abound in cancer, leading to over-proliferation of tumor cells and ineffective cancer therapy.^26,27^ Drugs including anti-CCL2 (C-C motif chemokine ligand 2), CSF1R (colony stimulating factor 1 receptor) antibodies suppress the TAM microenvironment’s potency surrounding the tumor are under investigation.^28^ Another way to improve the strength of CAR-T cells in solid tumors is to inhibit T-cell-mediated immune suppressor signals.^29^ Checkpoint inhibitors are therapeutic antibodies that bind to T-cell receptors. These antibodies can be useful as therapeutic strategies by hindering checkpoint molecules (such as CTLA4, PD-1) that cause T cell apoptosis. Results of clinical studies have shown that the expression of checkpoint inhibitors in CAR-T cells can be beneficial.^30,31^ A team of researchers fused MSLN-CAR-T cells with adenovirus expressing TNF-α (tumor necrosis factor-alpha) and IL-2 for the treatment of human and animal models that have improved the antitumor effects of engineered cells.^32^ Due to the limitations of therapeutic alternatives to cancer therapy and the effectiveness of CAR-T cells in various cancers, the removal of TME components that suppress the immune system’s role in treating different cancers may be a good option.

## Surface antigens

**Figure 3:**
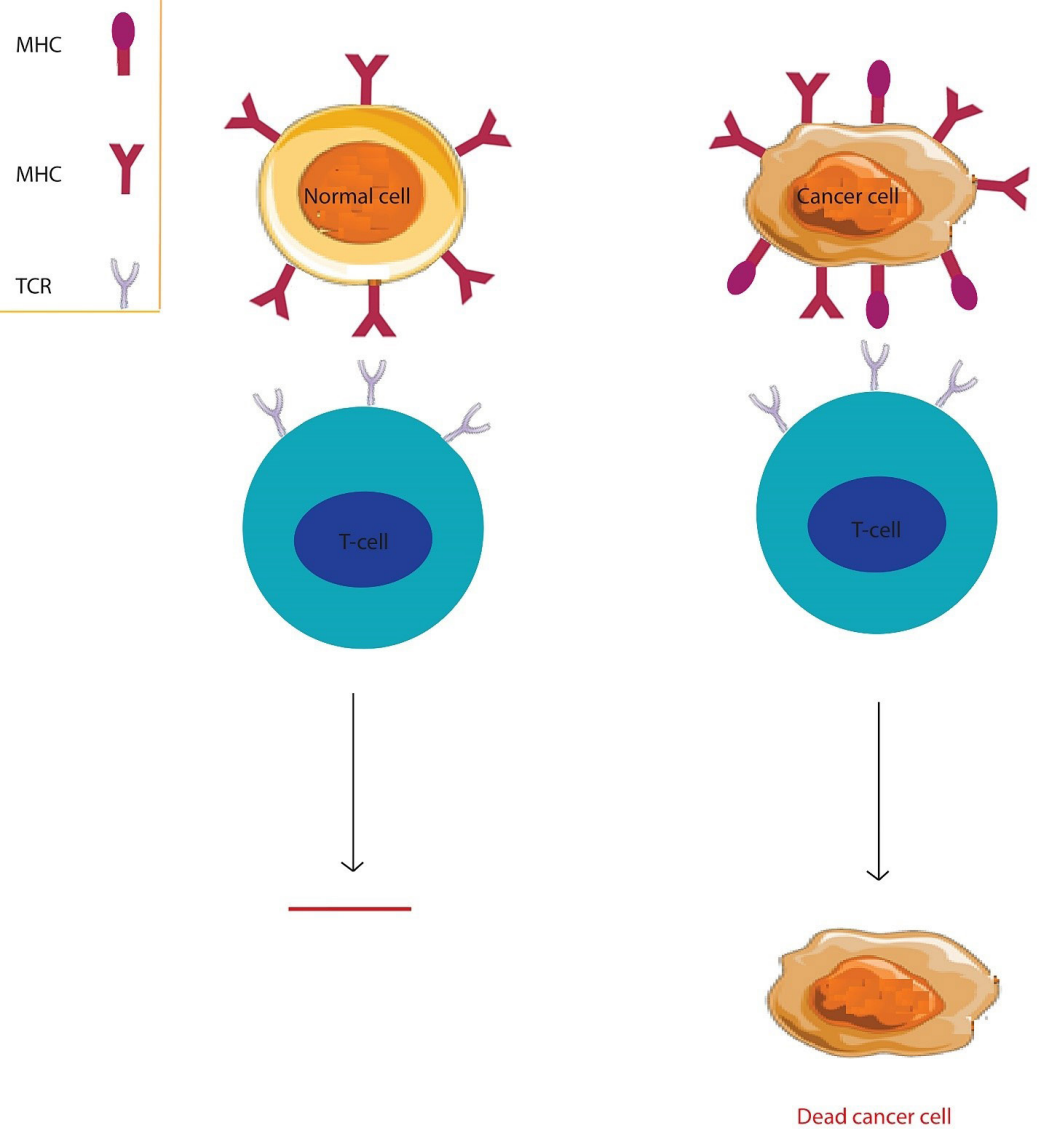
Targeting the right antigens to treat cancer. The expression of unique antigens in tumor cells and the lack of this antigen in normal cells reduces the extra-target toxicity and kills cancer cells.

Several properties have been introduced to target suitable antigens for therapy, particularly its unique expression in tumor cells and the lack of antigens in normal cells. Abnormal expression of specific antigens in multiple cancers suggests these antigens’ role in the survival, invasion, and metastases of these cells (Figure 3). Several CAR-T cell experiments targeting cancer cell antigen receptors, as shown in Table 1. Focusing on the high expression of these antigens in tumor cells over normal cells helps develop appropriate T cells with high affinity for cancer cells’ treatment. Several therapeutic target antigens have been studied for this purpose.

**Table 1:** Several experiments have demonstrated that CAR-T cells are characteristic of solid tumors

**Solid tumor**	**Antigen**	**Type of CAR-T cell**	**Property**	**conditions of research**	**Reference**
Pancreatic cancer	Mesothelin	meso-CAR-T cell	Improved performance; increasing the effect time of engineered cells	*In vivo*	32
Breast cancer	HER2	HER2-CAR-T cell	Contains CD28 or 4-1BB signal range for functional activity	*In vitro*	33
Glioblastoma	HER2	CAR-T Cell that target HER2 and IL-13Rα2	Improving performance with sequential expression; Cytokine secretion	-	34
Non-Small Cell Lung Cancer	EGFR	CAR-T Cell that target EGFR	Without severe toxicity, high tolerance for the patient	clinical study	35
Neuroblastoma	GD2 CD171	CAR-T Cell that target GD2 CE7-CAR T	Increase the anti-tumor effect; With central nervous system toxicity (CNS) No out-of-target toxicity	*In vivo* *In vivo*	36 37
Liver	CEA	CEA CAR-T	No side effects of grade 3 or 4	clinical study	38
Head and neck cancer	ErbB	CAR-T Cell that target ErbB(T1E28z)	Ability to target multiple antigens	clinical study	39
Prostate cancer	PSMA	PSMA- CAR-T Cell :Negative TGF-β Receptor	Increased cytokine secretion, resistance to burnout, long shelf life	clinical study	40

HER2: human epidermal growth factor receptor 2; EGFR vIII: Epidermal growth factor receptor variant III; EGFR: Epidermal growth factor receptor; GD2: is a disialoganglioside expressed on tumors; CD171: neural cell adhesion molecule L1; CEA: Carcinoembryonic antigen; ErbB: family of receptor tyrosine kinases; PSMA: prostate-specific membrane antigen.

### Mesothelin (MSLN)

A receptor is mainly present in mesothelial cells but increases in some human cancers such as ovarian, lung, and pancreas. Mesothelin (MSLN) can play a significant function in cell adhesion such that it can be studied in cell invasion.^41^ The expression of MSLN can be membrane or cytoplasmic in cancer cells. However, in mesothelial cancer cells, MSLN is also present in the cell membrane.^42^ In lung adenocarcinoma, the expression pattern of the MSLN is cytoplasmic and heterogeneous.^43,44^ Whereas cytoplasmic expression is more than membrane expression in gastric cancer.^45^ Due to high expression in certain cancers and low expression in normal cells, mesothelin may be the right candidate for immune cell engineering therapies.^46^ The concern about the MSLN CAR is the soluble MSLN, which could occupy the scFv segment. It should be noticed, however, that the activation of MSLN CAR-T cells depends on the presence of MSLNs in the cells. As a result, the toxicity of this treatment may be determined for normal cells expressing this receptor.^47,48^ The presence of soluble mesothelin-related peptide in serum does not change the effectiveness of MSLN CAR-T cells.^48,49^ The antigen deficiency of CAR-T cells is the target of the membrane due to the blockage of CAR by other serum proteins. This vulnerability can increase CAR-T cells by interacting with adhesion molecules and other by-products on T cells and tumor cells.^50^ Diacylglycerol kinase (DGK) is one of the TME immune cell inhibitors. If this inhibitory factor is removed, it can increase T cells’ proliferation and improve T cells’ effectiveness against cancer cells.^51,52^ Koretzky and colleagues have also demonstrated that DGKs elimination increases the activity of CAR-T cells against the tumor and stabilizes engineered cells targeting mesothelin receptors.^53^ CAR-T cells against MSLN could be effective against adenocarcinoma of the pancreatic duct. Intravenous (IV) transfer of these cells to animal models. Promising results from tumor shrinkage and even tumor degradation indicate that this receptor is a possible target for therapeutic options.^49^ In research performed by Beatty et al, due to high toxicity beyond the tumor to the production of CAR-T cells, mRNA-based methods have been used to reduce toxicity duration. Several injections were required to compensate for the short-term expression of CAR T. Out-of-tumor toxicity can cause adverse side effects through the use of engineered cells, as these target antigens are found at low levels in normal cells.^5^ Around 90 % of the folate receptor (FRa) and more than 65 % of the mesothelin receptor are expressed in ovarian cancer.^54,55^ Their pattern of expression in natural tissues is essentially non-overlapping. Therefore, Lanitis et al produced CAR-T cells that express all receptors simultaneously and reduce engineered cells’ potential toxicity to normal cells.^56^ MSLN-CAR T structures developed for activation of DAP12 killer immunoglobulin show improved *in vivo* potency compared to second-generation MSLN-CAR T with signaling domains.^57^ Adusumilli et al have demonstrated that intracellular administration of engineered immune cells displayed higher antitumor potency than intravenous injection.^48^ CAR-T cells are actively halted before the tumor enters the lungs. In summary, local infusion of MSLN-CAR-T cells enhances antigen exposure and boosts engineered cell activity.^42^

### Epidermal growth factor receptor

An intermembrane protein with tyrosine kinase activity can cause cell differentiation and proliferation. Increased expression of these receptors has been reported in many cancer types.^58,59^ Antibodies targeting EGFR include two groups: monoclonal antibodies targeting this receptor. These antibodies inhibit the action of the receptor by binding to the ligand-binding site. And the second category of inhibitors targeting tyrosine kinase activation in these receptors. These inhibitors prevent phosphorylation and prevent the transmission of the message to the cell. These antibodies were used to cure different forms of cancer, such as pancreatic cancer, kidney cancer, and colon cancer.^60,61^ In some patients, antibody treatment is not successful due to the various mutations in this receptor. It also seems essential to find the right treatments for these defects.^62^ One of the most common oncogenic mutations of EGFR is type VIII EGFR. This antigen tends to have all of the appropriate antigenic properties for CAR-T therapy since it is one of the most frequent EGFR mutations in cancers but has no expression in normal tissues.^63^ Glioblastoma (GBM) is the fatal component in adult brain tumors, and GBM treatment does not improve life span. EGFR VIII has a high degree of expression in the GBM cells. The targeting of this receptor by engineered cells to treat this cancer will also benefit.^64^ A team of researchers studied the ability of engineered cells to recognize EGFR VIII. With the help of CAR-T cells, they were able to target the EGFR VIII-containing GBM stem cells and identify the stem cells with this mutation.^65^ Although they do not respond to cultured normal tissue cells.^66^ EGFR-engineered immune cells have a significant anti-inflammatory effect *in vitro* and *in vivo*, but controlled and regulated toxicity such as skin, gastrointestinal, respiratory, and hypertension has been documented.^67^

### Human epidermal growth factor receptor

Glycoprotein is a member of the EGFR family. Overexpression of human epidermal growth factor 2 (HER2) has been shown in several cancers. This increased expression may mean that it is involved in the pathology of cancer. It is over-expressed in the breast and ovarian, and GBM cancers. However, this receptor is not present in normal brain cells and tissues.^68^ The elevated level of HER2 in different cancers has made it an appropriate target for CAR T therapy.^69^ Patients were treated with HER2 trastuzumab (herceptin) monoclonal antibody targeting the HER2 receptor. Observed in breast cancer treatment, Herceptin, which is also associated with chemotherapy, improves the lifespan of different stages of breast cancer.^70^ Haggett and colleagues have developed a CAR bispecific molecule targeting HER2 and IL-13Rα2 antigens in cancer cells. These antigens are found in cancer cells of the glioma. These engineered cells had successive expressions (TanCAR). This consecutive expression increases the function of the engineered cells and stimulates the secretion of cytokines. TanCAR-T cell agents can be used as an effective treatment strategy to regulate tumor development, but the detection of limitations for therapeutic use seems to be necessary.^34^ Clinical trials of tyrosine kinase HER2 inhibitors are progressing as low levels of HER2 are successfully identified in some cancers by Herceptin. This vulnerability has been used to treat some of these cancers.^71^ Results have indicated that 50% of patients have not undergone successful treatment and have developed resistance. This aversion leads to inefficiency in treatment.^72^

## CAR-T cells in hematological malignancies

Cancer cells use a variety of strategies to overcome the immune response of the body’s antitumor system. Immune evasion mechanisms include changes in the expression levels of checkpoint proteins in the cell cycle, increased expression of inhibitory receptors in immune cells such as PD-L1 (programmed cell death receptor), and Treg cell development.^73,74^ One of the defining characteristics of Treg cells is the aging of the immune cells, which in turn decreases the immune system’s capacity to fight cancer cells.^75^ These findings are obtained in hematological malignancies such as leukemia, multiple myeloma (MM), and B-cell lymphoma.^76,77^ They also provided different strategies to lead the immune system against malignant cells to evade cancer cells from the cell immune system (Table 2). For instance, T-cell injections that are genetically engineered and contain the expression of chimeric CD19-specific antigen receptors (CARs) could drive T-cells to CD19. Therapeutic experiments of CAR CD19 T cells were conducted with autologous patient cells involving substantial additional infrastructure.^78-80^ These results show appropriate protective strategies to fight hematological cancers.^81^ This treatment has undergone several trials and is considered an effective therapeutic regimen.^82^

**Table 2:** A variety of studies have demonstrated that CAR-T cells are characteristic of leukemia

**Disease**	**Target antigen**	**CAR features**	**Percentage of total rate**	**conditions of research**	**Reference**
DLBCL, MCL	CD19	CD8-alpha hinge and transmembrane domains	70%	clinical study	83
MCL, CLL	CD19 and CD20	Local is produced	< 60%	clinical study	84
B-cell ALL	CD19 and CD22	Bi-specific CAR; intracellular signaling domains 4-1BB and CD3ζ	< 80%	*In vivo*	85
DLBCL, MCL	CD19	Number of T cells enriched for central memory (A) and aimless memory (B)after CAR T injection on day 2	A = 100% B < 80%	clinical study	86
DLBCL, CLL	CD19	Express 4-1BBL	< 50%	clinical study	87
DLBCL,PMBCL, MCL, MALT	CD19alone(A) or CD19 plus another target(B)	Tumor biopsies stained for CD19, CD20, CD22, CD30, CD38, CD70, and PMSA. Choice of CAR T target based on staining results	Single A = 50% Double B > 90%	-	88
AML	CD123 and CD33	The presence of CD28OX40z increases the killing of CIK cells significantly	N/A	*In vivo*	89
AML	FRβ	Contains CD8a hinge domains and transmembrane domain with intracellular CD3z, alone or with CD28 signal range	N/A	*In vivo* *In vitro*	90
MM	BCMA	t has a CD137 (4-1BB) excitation motif and a CD3-zeta signal range	< 80%	clinical study	91

DLBCL: Diffuse large B-cell lymphoma; MCL: Mantle cell lymphoma; CLL: Chronic lymphocytic leukemia; B-cell ALL: B-cell acute lymphoblastic leukemia; PMBCL: Primary mediastinal B-cell lymphoma; MALT: mucosa-associated lymphoid tissue; AML: Acute myeloid leukemia; MM: Multiple myeloma.

### Multiple myeloma

MM is a type of blood cell cancer in which the plasma cells multiply and are highly abundant in the bone marrow. It accounts for around 10% of all malignancies in the blood. However, there are numerous treatments to cure this condition; it is intolerable.^75^ Engineered T-cell translocation is a therapeutic strategies approach to antigens. B-cell maturation antigen (BCMA) was a significant target for engineering cell studies.^92-94^ No expression of this target antigen has been identified in normal cells, although it is slightly expressed in normal and differentiated B cells. BCMA antigen is thought to boost the lifespan of MM cells and drug resistance in these cells.^95,96^ BCMA CAR-T cell therapy improves the intensity of response by more than 80%. However, the effect of treatment is temporary, and recovery has been documented after BCMA CAR-T cell therapy. The survival rate of BCMA CAR-T progression-free cells is approximately one year. Inhibition or suppression of BCMA expression may be an essential mechanism for disease progression that requires further investigation.^97,98^ Different approaches to BCMA in cell therapy, engineering cells for MM, are also being studied.^94^ Another target is CD138 (Syndecan 1), which is highly expressed in MM cells and is associated with disease pathogenesis. CD138 expression in MM cells in patients with progressive disease is higher than in MM cells in newly diagnosed patients. Implying the role of this antigen in the disease’s progression.^98^ However, given the broad expression of CD138 in human tissues, including epithelial cells, the targeted CARS of this antigen can be used with caution. For instance, treatment with BT062 causes skin and mucosal toxicity^99^ that shows the importance of integrating CD138 antigens with other target antigens in immune cell engineering. If we increase knowledge in this area, we are likely to discover more and better alternative approaches.

### Lymphoma

Lymphoma is present in the lymph nodes and leukemia in the bone marrow and blood. CAR) binds to CD19-targeted T cells to efficiently cure leukemia/lymphoma, thus decreases normal B cells. As a result, immunoglobulin replacement was necessary for the patient is over a lifespan, so the determination of an appropriate target was necessary.^100^ The CD30 antigen may serve as a proper target for engineered cells expressed in malignant lymphoid cells. It should be mentioned that while healthy lymphocytes still appear at certain stages.^101^ Due to its high presence in malignant cells, this antigen is a significant target for immune cell engineering antitumor malignancies that appears to be safe. Engineered cells targeting CD30 antigen show considerable anticancer activity in various models. However, targeting normal lymphocytes with CD30 antigen can cause severe complications for patients undergoing these therapies.^102,103^ During treatment with anti-CD30 CAR-T cells, the involuntary elimination of HSPCs resulted in unexpected outcomes. In addition to killing the lymphoma cells, the plasma of the blood cells has stabilized. This study observed that the best therapeutic index of anti-CD30 engineered T cells is appropriate for treating blood malignancies. They did not affect normal CD30 + HSPC.^100^

### Leukemia

More than 50 000 new leukemia patients and more than 20 000 leukemia-related deaths were documented in the United States in 2015.^104^ The patient’s life span is approximately five years, based on the type of leukemia, with the lowest survival associated with acute myeloid leukemia (AML) and the highest survival for acute lymphoblastic leukemia (ALL). Treatment of various chemotherapy methods due to drug resistance was not very satisfactory.^105^ Cancer immunotherapy has recently switched the therapeutic outlook as a useful clinical method for treating different types of cancer. Hematopoietic stem cell transplantation is among the immune-based treatments used for leukemia. Results in prolonged survival without disease symptoms in half of the patients with leukemia.^106^ However, this approach still has its limitations; for example, many patients relapse after treatment, which is not a reasonable option for many patients. The design of new, efficient, and safe treatment strategies for treating leukemia patients is also sensed. That new therapies are not appropriate for them.^107,108^

### B-cell leukemia

Significant advances in the treatment of leukemia have been accomplished by engineered cells. These CAR-T cells do this by targeting the antigen CD19.^107,108^ Even when antigens are found in both normal and cancer cells, most patients can tolerate long-term toxicity and survival^109^ and They demonstrate long-lasting longevity in the blood and bone marrow after injection.^108,109^ Injection of CD19-directed CAR-T cells to ALL patients who are resistant to treatment or relapse has a response rate of over 70%.^110,111^ Although CD 19 acts as an alternative to the treatment of all engineered cells, research has shown that toxicity is beyond the scope of a potential barrier to engineered immune cells for treatment. As a result, additional optimization is necessary for the identification of targets.^112^ The CD22 antigen was evaluated as a therapeutic target for CAR-T cell immunity to destroy leukemia cells. Lymphoblasts isolated from patients with precursor B cells all showed CD22 expression.^113^ Cells engineered with CD22 antigens, such as CAR-T anti-TSLPR cells, repeatedly lyse target B cells under different environmental conditions.^114^ The 4-1BB domain involvement causes the toxicity of engineered immune cells, while previous studies have documented the beneficial function of 4-1BB domains in CAR-T cells. This inconsistency may be attributable to discrepancies between the structure’s configuration and the tumor model.^115,116^ Another research has shown that altering the position of immunoglobulins in engineered immune cells decreases the antitumor effect.^114,117^

### Myeloid leukemia

Lately, CAR-T therapeutic strategies have focused on AML leukemia, the most prevalent form of myeloid neoplasm in adults.^118,119^ Hematopoietic stem cells (HSPCs) are thought to be an effective treatment for this disease. However, the mortality and other implications of this treatment indicate the vulnerability of this treatment. Detailed research towards using engineered immune cells is appropriate to solve problems in CAR T and offer appropriate therapeutic strategies. CD123 myeloid antigen is one of the potentially promising targets that are undergoing study. While CD123 antigen is present in HSPC cells to a limited extent, it has been well-tolerated when designed to target antigen for AML patients’ treatment. Treatment with engineered cells may decrease the burden of AML at the advanced stage of the disease and suggest that treatment may be effective at various stages of the disease.^89^ The comparison between CD123 CAR-T and CD33 revealed that CD123 CAR-T was less toxic to regular hematopoietic stem cell activity. However, an independent clinical trial in xenograft models showed CD123-targeted CAR-T cells’ effect on normal bleeding.^120^ The next target is the folate receptor (FR), a glycoprotein with a large cysteine amino acid. This receptor contains isoforms (FRa, FRb, and FRc) and facilitates folic acid absorption. The FRb isoform is often expressed in myeloid hematopoietic cells also expressed in different malignancies.^121^ It has recently been shown that engineered cells targeting human FRb receptors can detect and kill target cells.^90^ The combination of CARB-FR and FRb with trans-retinoic acid increases FRb levels and antitumor activity. Notably, FR-targeted cells do not have a detrimental effect on hematopoietic stem cells *in vitro*. The same group has demonstrated that HA-FRb engineered cells destroy cancer cells more quickly than previously engineered cells.^122^

### Chronic lymphocytic leukemia

CLL is a chronic malignancy of lymphocytes common in adults.^123^ Allogeneic stem cell transplantation is the only current CLL therapy approach.^124^ Patients with chronic and high-risk CLL are treated with CD19 CAR-T cells.^111^ Several studies have been reviewed to evaluate the effect of engineered cells on individuals with CLL. Even though CLL pathogens contribute to premature immunodeficiency, the impact of CAR-T cells in CLL patients would be restricted. Appropriate approaches to the above problem need to be identified.^125^

### T-cell leukemia

Application of immunotherapy for T-cell neoplasms has been investigated.^126,127^ Interestingly, the T lymphocytes’ cancer cells and regular lines have the same antigens, and no specific antigens have been identified to distinguish them. The use of CAR-T cells will also kill normal cells. Despite these issues, the evaluation of CAR-T cells designed to attack CD5 antigens was evaluated. This study’s findings revealed that engineered cells are attracted to cells that express CD5 and kill these cells. Normal T cells with unregulated expression of PI-9, cathepsin B, and BCL-2 have also been shown to prevent cell death against engineered immune cells.^126^ They are also designed to attack CD4 antigens; CAR-T cells strongly destroy CD4 + leukemia cell lines *in vitro* lymphoma models and *in vivo* T cells.^127^ These findings suggest that engineered cells can provide appropriate therapeutic approaches but need many experiments.

## Limitations

**Figure 4:**
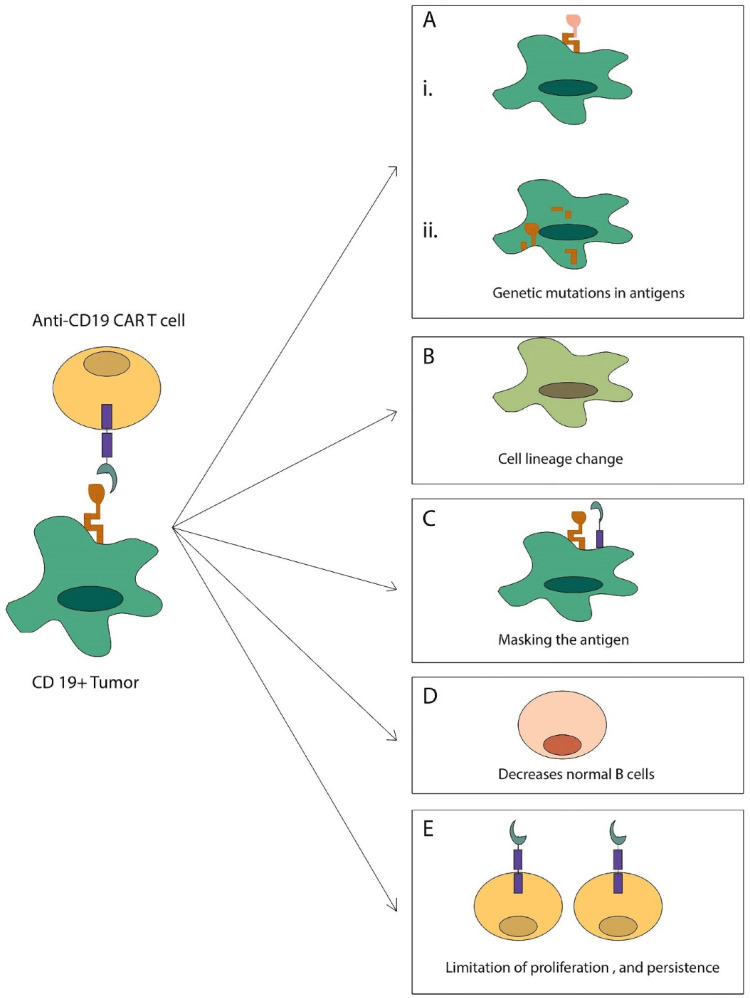
Restrictions on the use of CAR-T. (A) (i) the cancer cells alter CD19 receptor expression so that other CAR-T cells are unable to identify and bind to the target, or (ii) they completely lose CD19 receptor expression. (B) Tumor cells can transform phenotypic deformation into a different breed that is inherently CD19 negative. (C) Cancer cells can cover the surface of the epitope and deprive them of access to CAR-T cell cells. (D) Cause off-target toxicity. (E) CAR-T cells have limited proliferation and persistence. And after a short time, they disappear.

Cancer treatment has achieved a new phase with the shift from chemotherapy to safety-based therapeutic approaches. In the meantime, CAR-T cells have demonstrated considerable therapeutic potential in cancer therapy. Although the treatment of CAR-T cells in cancer was very successful, it is still in its infancy, and the mechanisms of action of CAR-T cells are not fully understood. There are more limitations to CAR-T cell therapy that has not been removed. These limitations in the treatment of cancer CAR-T cells include (a) limitation of T cell function, proliferation, and persistence, (b) Restriction of CAR-T cell importation to the tumor site; (c) extra-target toxicity, and (4) loss of antigen to tumor cells, which causes the tumor to escape from CAR-T cells (Figure 4).^29,128^ ScFv-based CAR-T cells could be directed to cancer cell antigens if the antibody’s amino acid sequence with the target properties is identical. However, their use in CAR-T cells can be limited.^19^ For instance, by comparing two anti-CD19 (FMC63-scFv) or GD2 (14g2a-scFv) CAR, it was found that 14g2a-scFv structure domains establish clusters independent of the anti-GD2-CAR antigen on the T cell surface. As a result, it promotes severe anti-GD2-CAR-T cells’ severe weakness and reduces their ability to act appropriately *in vivo*. Many CAR clinical trials have used scFv derived from mouse antibodies to increase the probability of anti-CAR-T cell response, which may cause toxicity or reduce T cells’ viability.^129,130^ This problem can be overcome by humanizing mouse scFvs or removing scFv from fully human antibodies.^131,132^ Human CAR-T cells can induce comparative recovery of ALL resistant to previous anti-CD19-CAR therapeutic cells based on scFv mice.^132^ However, due to the receptors’ chemical existence, even human protein-derived structures can induce host immune response.^18^ It is doubtful that the current CAR design template would enable T cells to resolve the multiple obstacles presented by tumor cells and the tumor’s microenvironment. The intrinsic defects of CAR-T cells must therefore be compensated for and novel techniques developed. Targeting more than one antigen on tumor cells at the same time can prevent the loss of the antigen and the tumor from escaping.^26^ Due to tumorigenesis’s complex nature, even optimized cars cannot resolve all the various tumor entities’ obstacles. It is also essential to combine several modifications to compensate for some of the inherent defects of CAR-T cells and fulfill the specific needs of tumor cells and the microenvironment. To produce the best therapeutic outcomes, T cell trafficking, penetration, and persistence must be optimized.

## Conclusion

TME significantly inhibits T-cells’ function and indicates that engineered cells may be less effective in targeting tumor cells. However, new therapeutic strategies for CAR-T cell repair and recovery and CAR-T cell activation also have promising approaches to modulate signaling at the tumor cells, transforming the immunosuppressive microenvironment into an efficient microenvironment. CAR-T cells in solid tumors require further investigation. Further research is needed for CAR-T cells in solid tumors. The potential insights into immunotherapy can be provided by understanding the cancer outbreak. Therapies are also different because cancer mechanisms differ.

## Ethical Issues

This article does not contain any studies with human participants or animals performed by any of the authors.

## Conflict of Interest

No potential conflict of interest was reported by the authors.
